# Elucidation of Antiviral and Antioxidant Potential of C-Phycocyanin against HIV-1 Infection through *In Silico* and *In Vitro* Approaches

**DOI:** 10.3390/antiox11101942

**Published:** 2022-09-28

**Authors:** Pratiksha Jadaun, Chandrabhan Seniya, Sudhir Kumar Pal, Sanjit Kumar, Pramod Kumar, Vijay Nema, Smita S Kulkarni, Anupam Mukherjee

**Affiliations:** 1 ICMR-National AIDS Research Institute, Pune 411026, MH, India; 2 Meerut Institute of Engineering & Technology, Meerut 250005, UP, India; 3 Vellore Institute of Technology, Vellore 632014, TN, India; 4 ICMR-National Institute of Cancer Prevention and Research, Noida 201301, UP, India

**Keywords:** C-phycocyanin, phycobiliprotein, marine algae, reactive oxygen species, cytotoxicity, anti-HIV-1 activity, anti-retroviral

## Abstract

Antiretroviral therapy is the single existing therapy for patients infected with HIV; however, it has drawbacks in terms of toxicity and resistance. Thus, there is a continuous need to explore safe and efficacious anti-retroviral agents. C-Phycocyanin (C-PC) is a phycobiliprotein, which has been known for various biological properties; however, its effect on HIV-1 replication needs revelation. This study aimed to identify the inhibitory effects of C-PC on HIV-1 using *in vitro* and *in silico* approaches and to assess its role in the generation of mitochondrial reactive oxygen species (ROS) during HIV-1 infection. *In vitro* anti-HIV-1 activity of C-PC was assessed on TZM-bl cells through luciferase gene assay against four different clades of HIV-1 strains in a dose-dependent manner. Results were confirmed in PBMCs, using the HIV-1 p24 antigen assay. Strong associations between C-PC and HIV-1 proteins were observed through *in silico* molecular simulation-based interactions, and the *in vitro* mechanistic study confirmed its target by inhibition of reverse transcriptase and protease enzymes. Additionally, the generation of mitochondrial ROS was detected by the MitoSOX and DCF-DA probe through confocal microscopy. Furthermore, our results confirmed that C-PC treatment notably subdued the fluorescence in the presence of the virus, thus reduction of ROS and the activation of caspase-3/7 in HIV-1-infected cells. Overall, our study suggests C-PC as a potent and broad *in vitro* antiviral and antioxidant agent against HIV-1 infection.

## 1. Introduction

Current antiretroviral drugs for HIV-1 afford long-term and sustainable treatment options, allowing HIV-infected individuals to survive for many years. However, even fully suppressive antiretroviral therapy (ART) does not clear HIV-1 from the host and cessation of ART often leads to viral rebound [1]. Suppression of latent HIV-1 reservoirs requires life-long ART and that can be associated with serious complications, including cardiovascular and renal failure, peripheral neuropathy, and neurologic disease. Chronic antiretroviral drug treatment also promotes acquired drug resistance, leading to decreased ART efficacy and the potential spread of drug-resistant viruses [2]. Recent estimates report 1.5 million new HIV infections worldwide, totaling up to 37.7 million people living with HIV (UN-AIDS 2021). Controlling the spread of HIV infection with efficient management is therefore considered a top priority. These observations underscore the need for novel strategies to suppress HIV-1 replication and eliminate persistent viral reservoirs to achieve a functional cure.

Natural products are identified as the new remedial agents for the management of infectious diseases and they have been considered one of the traditional key strategies for the advancement in the field of medicines [3]. One of the key pigment ingredients of Spirulina is phycocyanin, which is made up of oligomers and has α and β subunits, the αβ-pairs mostly build the pigment as a trimer (αβ)3 or hexamer (αβ)6. Both α and β subunits have a bilin chromophore, which contains linear tetrapyrrole rings that are attached to the cysteine amino acid of the apoprotein by thioether linkages [4,5].

In many countries, phycocyanin and other components of Spirulina are used as a dietary supplement with well-documented nutritional benefits [6,7,8]. There is evidence that in the Mayan and Aztec civilizations and from communities in Central Africa, Spirulina was harvested for use as food [9,10]. The water-soluble pigments of Spirulina such as C-Phycocyanin are currently the subject of extensive research to determine their potential for use in medicine and therapy [11,12], in addition to its importance as a food additive. C-Phycocyanin has been studied extensively through *in vitro* and *in vivo* research for its antioxidant activity, which includes the ability to activate antioxidant enzymes, scavenge free radicals, and protect against lipid peroxidation and DNA damage [6,13,14]. Extracts of Spirulina have been reported to have multiple therapeutic effects, including cholesterol reduction, immunomodulation, antioxidant, anti-cancer, and anti-viral effects [15,16].

In the HIV-1 life cycle, the virus uses three important enzymes for its replication, including Integrase (HIV-1-INT), Reverse Transcriptase (HIV-1-RT), and Protease (HIV-1-PR) [17]. Thus, the blocking of these enzymes can inhibit viral replication and is an important strategic approach for anti-HIV drug discovery. Now, many HIV-1 inhibitors are available, but the adverse effects of these drugs are being counted [18]. Consequently, the discovery of novel compounds from natural sources is important to research for controlling HIV-1 infection. Therefore, antioxidant activity along with added effects of phycobiliprotein, C-Phycocyanin might be an imperative asset of medicine. Notably, the effect of this pure protein has not been investigated extensively in anti-HIV-1 research. In this study, we conducted cellular assays to examine the effects of C-Phycocyanin upon the pathogenicity of several HIV-1 strains and identified the underlying mechanisms involved, furthermore, determined its role as a free radical scavenger during HIV-1 infection.

## 2. Materials and Methods

### 2.1. Phycobiliprotein C-Phycocyanin

Phycobiliprotein, C-Phycocyanin (C-PC) a novel protein from *Spirulina sp.* was purchased commercially (Sigma-Aldrich, St. Louis, MO, USA). The working stock of C-PC (2 mg) was used for assessing the cell cytotoxicity and performing anti-HIV-1 testing.

### 2.2. Protein Structure Retrieval and Preparation for Docking Simulations

The 3D C-PC (PDB ID: 1HA7), HIV-1-RT (PDB ID: 1C0T), HIV-1-PR (PDB ID: 5KR0), HIV-1-INT (1QS4), HIV-1 glycoprotein GP120 (37J0), host co-receptors CxCR4 (3ODU) and CCR5 (4MBS) were retrieved from Research Collaborator for Structural Bioinformatics (RCSB) Protein Data Bank (PDB) [19]. The optimal 3D structure were obtained after cleaning of protein complex using CHIMERA 1.16 and the stereo-chemical quality check and energy minimization was performed in Swiss-PDB viewer [20,21]. HADDOCK v2.4 server was used for protein–protein docking and the results were analyzed using PyMol 2.5.2 and Protein-Ligand Interaction Profiler [22,23,24]. Based on the average score of the top four individuals in each cluster, the clusters were ranked.

The score was calculated as:

$${\mathrm{HADDOCK}}_{\mathrm{score}}=1.0{\;*\;E}_{\mathrm{vdw}}+0.2{\;*\;E}_{\mathrm{elec}}+1.0{\;*\;E}_{\mathrm{desol}}+0.1{\;*\;E}_{\mathrm{air}}$$

According to Fernandez-Recio et al., 2004, E_vdw_ stands for the intermolecular Van der Waals energy, E_elec_ for the intermolecular electrostatic energy, E_desol_ for an empirical desolvation energy factor, and E_air_ for AIR energy [25].

For cross-verification and benchmarking of our results obtained using the above method, we used Molecular Operating Environment (MOE) software for preparing the proteins, finding pockets for receptor protein, identification of active sites and grid preparation and finally docking the C-PC with HIV-1 RTase (1C0T) and Protease (5KR0) [26]. Additionally, we examined the molecular interactions between C-PC and other viral proteins such as Integrase (1QS4), glycoprotein GP120 (37J0), Host co-receptor CxCR4 (3ODU) and CCR5 (4MBS) using the same *in silico* tool. The detailed methods are available in the Supplementary File as Appendix A.

### 2.3. Cell Lines and HIV-1 Stock

TZM-bl cells (HeLa modified cell line; initially called JC53-bl; clone 13) were procured from the National Institute of Health (NIH)–HIV Reagent Program and maintained in DMEM (Gibco, MA, USA) containing 10% FBS (Moregate, Bulimba, QLD, Australia) and supplemented with HEPES (Gibco, Waltham, MA, USA), antibiotics (Sigma-Aldrich, St. Louis, MO, USA) at 37 °C in a 5% CO_2_ humidified chamber. At least 80% of confluent cells were used for any further experiments in this study. The peripheral blood mononuclear cells (PBMCs) were separated from the blood of healthy individuals through density gradient centrifugation with Histopaque (Sigma-Aldrich, St. Louis, MO, USA) and were activated with PHA-P (5 µg/mL) (Sigma-Aldrich, St. Louis, MO, USA) in the complete medium of RPMI 1640 (Gibco, Waltham, MA, USA) containing 10% FBS and 5U/mL Interleukin-2 (IL-2) as growth factor (Sigma-Aldrich, St. Louis, MO, USA). The activated PBMCs were used for HIV-1 stock development and confirmation of anti-HIV-1 activity.

The viral isolates HIV-1_92/RW/008_ (R5, Subtype A), HIV-1_Ada5_ (R5, subtype B), HIV-1_VB051_ (R5, Subtype C) and HIV-1_UG070_ (X4, Subtype D) were obtained from the virus bank repository maintained at the Division of Virology, ICMR-National AIDS Research Institute, Pune. The virus stocks were prepared in PHA-P activated PBMCs quantified by HIV-1 p24 antigen detection assay (Abcam, Cambridge, UK). Titration of these viral stocks was made in the TZM-bl cell lines, and the TCID_50_ (i.e., 50% of tissue culture infectivity dose) was determined using the Spearman Karber method [27].

### 2.4. Cytotoxicity Assay by MTT

The cytotoxicity of the C-Phycocyanin to TZM-bl cells and activated PBMCs was determined using the MTT assay (Sigma-Aldrich, St. Louis, MO, USA) as described previously [28]. Briefly, double dilutions of C-PC working stock (2 mg/mL) were prepared and added onto 1 × 10^4^ adherent TZM-bl cells/well seeded in 96-well plates kept for overnight incubation under 5% CO_2_ at 37 °C. After 48 h post-treatment, the cell viability was determined using 20µL (5 mg/mL) MTT reagent. Similarly, the activated PBMCs were treated with various concentrations of the pure protein and incubated for 5 days with 5% CO_2_ at 37 °C incubator. Subsequently, the cell viability was determined using the MTT reagent as described above. After the final incubation, the optical density (OD) value was recorded at 550 nm and 630 nm using a multimode plate reader. The CC_50_ values were obtained at the concentration where 50% of the cells remain viable in the presence of C-PC.

### 2.5. Cell Associated Anti-HIV-1 Assay

Based on the cell viability assay, a range of non-cytotoxic concentrations of the C-PC protein were selected and the anti-HIV-1 activity was evaluated. In a 96-well plate, the TZM-bl cells (1 × 10^4^ cells/well) were first infected with different HIV-1 strains (subtype A-D) for 2 h at 37 °C in a 5% CO_2_ atmosphere before exposing the infected cells to the serial dilutions of C-PC in duplicate (0.500–0.031 mg/mL). At 48 hpi, the luciferase activity was measured using the Britelite plus reagent (Perkin Elmer, Waltham, MA, USA). A reverse transcriptase inhibitor, azidothymidine or AZT, was taken as a positive control compound with anti-HIV-1 activity in the experiment.

### 2.6. HIV-1 p24 Antigen Capture Assay

The PBMCs were seeded in 96-well plates (0.2 × 10^4^ cells/well) and infected with HIV-1_VB051_ (40 TCID_50_). This was followed by the addition of non-toxic concentrations of C-PC (0.5–0.195 mg/mL). After incubation for 5 days at 37 °C in a 5% CO_2_ incubator, the virus supernatant was analyzed for the presence of HIV-1 p24 antigen through ELISA following the manufacturer’s instruction (Abcam, Cambridge, UK). The absorbance was measured at a wavelength of 450 nm in an ELISA plate reader (BioRad, Hercules, CA, USA) and 50% inhibition concentration or IC_50_ values were calculated based on the positive control provided in the kit.

### 2.7. HIV-1 Reverse Transcriptase Activity Assay

The inhibitory action of C-PC on HIV-1-RT was assessed using a commercially available kit (Roche, Mannheim, Germany). Briefly, dilutions of the C-PC were incubated with the HIV-1 RTase in addition to a template nucleotide mixture for 1 h. Consequently, the mixture was transferred to the microwell plates coated with streptavidin and followed by incubation for 1 h to bind with biotin, which is a DIG-labeled template primer complexed to the streptavidin-coated plate. The enzyme HRP conjugate was added to the above plate and followed by 1 h incubation. After the addition of the substrate, the color responses were measured at wavelengths 405 nm and 490 nm as a reference wavelength using BioRad reader PR4100. The IC_50_ values were determined by non-linear regression curves. A known reverse transcriptase inhibitor nevirapine (NVP) was used as positive control.

### 2.8. HIV-1 Protease Assay

The inhibition of the HIV-1 protease enzyme by C-PC was examined using a commercially available kit following the manufacturer’s instructions (Sigma-Aldrich, St. Louis, MO, USA). A known protease inhibitor Ritonavir was used as positive control.

### 2.9. Detection of Intracellular Reactive Oxygen Species (ROS)

To quantify the formation of ROS in TZM-bl cells, the MitoSOX molecular probe was used and the assay was performed as described earlier [29]. Briefly, TZM-bl were seeded into 96-well plates at a density of 1 × 10^4^ cells per well in a volume of 100μL complete medium. After overnight incubation, cells were infected with the virus and treated with C-PC at different concentrations (0.500–0.031 mg/mL), and the assay was performed at 24 hpi. Initially, the infected cells were treated with 10μM MitoSOX at 37 °C in 5% CO_2_ incubator. At 30 min post-incubation, the fluorescence intensity was measured at an excitation wavelength of 485 nm and an emission wavelength of 528 nm using a microplate reader (Perkin Elmer, Waltham, MA, USA).

### 2.10. Confocal Microscopy

A MitoSOX red molecular probe was used to identify the intracellular ROS production as superoxide (O_2_^•−^) using confocal microscopy as described earlier [30]. Briefly, the TZM-bl cells were seeded onto the glass coverslips in a 6-well plate and after treatment for 24 h, the cells were incubated for 30 min with MitoSOX (10 μM) in the dark atmosphere. Subsequently, after washing with PBS, the cells were fixed with 3.7% paraformaldehyde and visualized under the laser-scanning confocal microscope (Nikon, Tokyo, Japan). Known ROS generators, 0.1 mM Xanthine + 0.01 U Xanthine oxidase were used as the positive control. 100U Cu–Zn SOD was also used as an inhibitor of ROS.

Similarly, intracellular ROS generation was also detected using 2′,7′-dichlorofluorescein diacetate DCF-DA (10 µM) green molecular probe using confocal microscopy as described earlier [31].

### 2.11. Screening of Caspase Activity

The Caspase-Glo 3/7 Assay (Promega, WI, USA) was used for the detection of *in vitro* caspase activity in the HIV-1-infected C-PC treated cells. The caspase-3/7 reagent contains the DEVD, a tetrapeptide aminoluciferin substrate, known for the detection of caspase-3/7 and luciferase activity. The addition of Caspase-Glo 3/7 directly onto the cells results in cell lysis followed by the caspase cleavage by the DEVD substrate, hence luminescence generation. The quantity of luminescence in the readout is relative to the amount of caspase activity present in the cells.

### 2.12. Assessment of Cell Death through FACS

The Annexin V-FITC conforming signal delivers a precise sensitive technique for detecting cellular apoptosis, whereas propidium iodide (PI) is used to detect necrotic or late apoptotic cells, categorized by the loss of the plasma and/or nuclear membranes integrity. Hence, cellular apoptosis was detected in the HIV-1-infected C-PC treated cells stained with Annexin V-FITC/PI through flow cytometry analysis. The Annexin V-FITC binding was recorded by the FL-1 signal detector, while the PI staining through the emission of FL-2 signal detector using BD FACS Vantage Analyzer (Becton–Dickinson, NJ, USA). A total of 10,000 events were acquired for the determination of fluorescence intensity and the Cell Quest software was used for the data analysis. Camptothecin (15 µm) was taken as a positive control for the FACS analysis.

## 3. Results

The present study aimed to evaluate the anti-HIV-1 and antioxidant activity of the major phycobiliprotein present in many marine cyanobacteria or some blue-green algae, known as C-Phycocyanin or C-PC, against the multiple subtypes of HIV-1 for anti-retroviral drug screening.

### 3.1. Cytotoxicity of C-Phycocyanin

**Figure 1:**
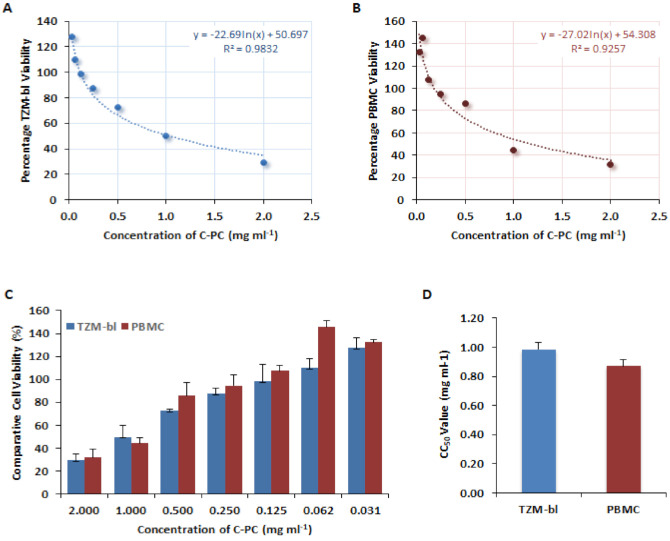
Determination of cytotoxic concentration of C-Phycocyanin. The effect of different concentrations of C-PC on (**A**) TZM-bl cells and (**B**) PBMCs. (**C**) The comparative graphical illustration of viability between TZM-bl cells and PBMCs at 2.0–0.0312 mg/mL concentrations of C-PC. (**D**) The mean CC_50_ values of C-PC as determined TZM-bl and PBMC from three independent assays.

The C-PC was screened to assess its effect on the viability of TZM-bl cells and PBMCs by MTT-based quantitative and colorimetric assay. The dose-dependent effect of C-PC (2.0–0.0312 mg/mL) was represented by the concentrations against the percentage of cell viability (Figure 1A,B). The comparative cell viability between TZM-bl and PBMCs was depicted as almost analogous (Figure 1C). The concentration that allows 50% viability of the cells was represented as CC_50_ value found to be ≥0.986 and ≥0.872 mg/mL for TZM-bl and PBMC, respectively (Figure 1D). The data showed that C-PC was safe and non-toxic to the cells in a range of 0.5000–0.3125 mg/mL for treatment, and was used in subsequent studies.

### 3.2. Anti-Viral Activity of C-Phycocyanin against HIV-1

**Figure 2:**
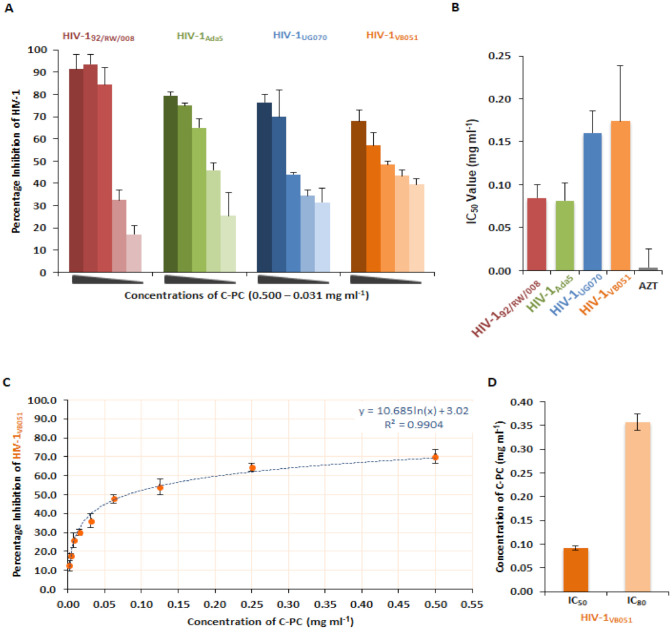
C-PC inhibits HIV-1 replication. (**A**) Percentage inhibition of four different subtypes of HIV-1 replication in the presence of C-PC (0.500–0.0312 mg/mL) in TZM-bl cells. (**B**) The IC_50_ values for HIV-1_92/RW/008_, HIV-1_Ada5_, HIV-1_UG070_, and HIV-1_VB051_ in TZM-bl. (**C**) Dose-dependent activity of C-PC in the PBMCs infected with HIV-1_VB051_. (**D**) IC_50_ and IC_80_ concentrations of C-PC in HIV-1-infected PBMC. Standard drug AZT was used as the positive control of HIV-1 inhibition.

We examined the antiviral activity of C-Phycocyanin via luciferase gene assay in TZM-bl cells. In the cell-based assays, TZM-bl cells were infected with four different subtypes of HIV-1 strains, individually, prior to the addition of different concentrations of C-Phycocyanin and incubated for 48 h before the termination of the assay. Treatment of C-PC at different concentrations (0.3125–0.500 mg/mL) led to comparable dose-dependent inhibition of HIV-1_92/RW/008_ (subtype A), HIV-1_Ada5_ (subtype B), HIV-1_VB051_ (subtype C) and HIV-1_UG070_ (subtype D) virus strains (Figure 2A). The 50% inhibition concentration or the IC_50_ values were determined as 0.0846 mg/mL, 0.0817 mg/mL, 0.1603 mg/mL, and 0.1740 mg/mL for HIV-1_92/RW/008_, HIV-1_Ada5_, HIV-1_UG070_, and HIV-1_VB051_, respectively (Figure 2B). Consequently, the anti-HIV-1 activity of C-PC was further confirmed in the PBMCs using the primary isolate HIV-1_VB051_, and a comparable pattern of dose-dependent inhibition of HIV-1 p24 antigen was determined (Figure 2C). The IC_50_ value detected for C-PC in PBMC was 0.090 mg/mL (Figure 2D). Even the 80% inhibition of HIV-1 infection was observed at the non-toxic concentration of 0.3566 mg/mL of C-PC as recorded by the IC_80_ value (Figure 2D). Together, these results demonstrated that C-PC is capable of inhibiting viral replication against a broad range of HIV-1 clades.

### 3.3. Interactions of C-PC and HIV-1 Reverse Transcriptase

Computational studies were used to understand C-PC and HIV-1RT protein–protein interaction by performing Molecular Docking Simulation using the HADDOCK4.2 server. The molecular interactions were analyzed by measuring distances between protein residues, analyzing hydrogen bond and Van der Waals interactions, and binding energies, as shown in Table 1. Briefly, a hydrogen bond was defined as having a donor-hydrogen acceptor angle of more than 120° and a distance between the donor and acceptor of <3.5 Å.

**Figure 3:**
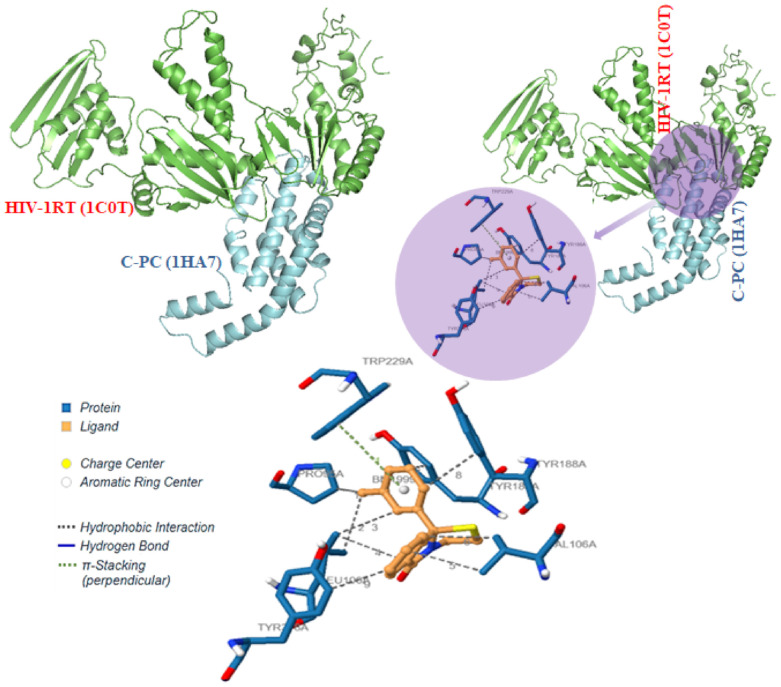
Molecular interactions between C-PC (1HA7) with HIV-1 Reverse Transcriptase (1C0T). HIV-1RT (green) active residues as well as other important residues are occupied by C-PC (aqua blue) and therefore inhibit the reverse transcription activity.

The p66 polymerase in HIV-1RT has a right-handed structural organization that contains finger subdomain residues (1–84, & 120–150), palm subdomain residues (85–119, and 151–243), and thumb subdomain residues (244–322), and liking subdomain residues (323–427) such as other polymerases. The polymerase active site residues (Asp110, Asp185, and Asp186) are found in the β6- β10- β9 sheet of the palm subdomain, and all these domains define a primer binding cleft [32]. It is carboxylated in the p66 palm subdomain, which interacts with two magnesium divalent ions (Mg^2+^) for catalysis and, as a result, aids in the addition of nucleotides to the developing primer strand known as “primer grip” for proper positioning of the 3′ end [33]. Tyr-Met-Asp-Asp is present in the subunits p66 and p51, but the “thumb’s knuckle” is close to Trp239 (positions β14 and β15) and Val317 (position β15). The β5– β6 of the loop, β6, β9–10, β12– β13 of the hairpin, β15 of the p66, and β7– β8 connecting loop of the p51 make up the majority of the binding pocket for non-nucleosides for inhibition. Gly231, Trp266, Tyr188, and Trp229 side chain residues are also present in this pocket [32]. The protein–protein molecular modeling and simulation studies revealed molecular alignment of C-PC with HIV-1RT by forming three hydrogen bonds with active residues Leu279 (3.03 Å), Lys281 (3.03 Å) and Lys281 (2.55 Å) (Table 2) and 09 hydrophobic interactions with Tyr181, Tyr188 and Tyr318 along with other sub-site catalytic residues Pro95, Leu100, Lys101, Lys103, Val106, Glu138, Val179, Ile180, Gly190, Trp229, His235, and Tyr310 in HIV-1RT (Table 2 and Figure 3). The active site catalytic residues, motif, and other noteworthy residues mentioned above located in the active pocket of HIV-1RT are occupied by C-PC, therefore playing a role in the inhibition of HIV-1-RT activity [34].

The S-value obtained for C-PC and HIV-1RT interaction using MOE docking with a best score of −80.38 with the RMSD value at 1.105. The other scores obtained with reiteration are as given in Table S1. Amino acid Met69A of C-PC was found to have hydrophobic interaction with Met230 of HIV-1RT with a distance of 3.591Å while Asp77A of C-PC was shown to have ionic interaction with Lys259 of 4.456Å. Furthermore, Gln70A and Thr21B of C-PC were found to have contact interaction with Gly262 and Arg448 residues of HIV-1RT (Tables S2 and S3, Figure S1).

### 3.4. Inhibitory Effect of C-Phycocyanin on HIV-1 Reverse Transcriptase

**Figure 4:**
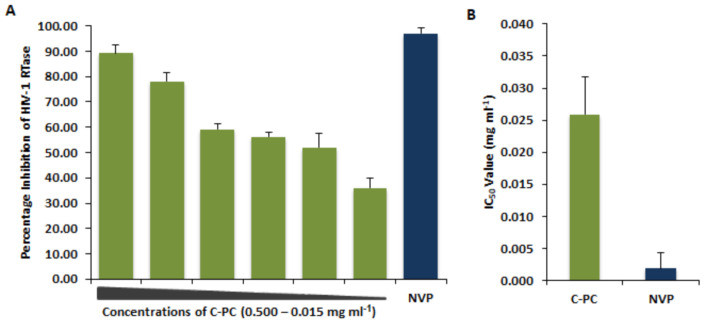
C-PC inhibits HIV-1 viral enzyme Reverse Transcriptase. (**A**) Percentage inhibition of HIV-1 RTase enzyme activity in the presence of C-PC in a dose-dependent (0.50–0.15 mg/mL) assay. A known HIV-1 RT inhibitor NVP (2 μM) was used as positive control. (**B**) Comparative analysis of IC_50_ concentrations of C-PC and NVP on HIV-1 RTase activity.

The HIV-1 RT is a unique and key enzyme that controls HIV-1 replication in the infected cells. In this study, we found that the increasing concentrations of C-PC (0.015–0.500 mg/mL) inhibited the activity of HIV-1_RT in agreement with the *in silico* studies. Whereas the maximum inhibition of HIV-1 RTase (89.8%) was observed at the highest concentration of 0.500 mg/mL (Figure 4A), the IC_50_ value was found to be 0.025 mg/mL for C-PC protein (Figure 4B). The results were compared with the known RTase inhibitor NVP, at a concentration of 2μM for 97% inhibition of the viral protein, with the IC_50_ value found to be at 0.0018 mg/mL (Figure 4B).

### 3.5. C-Phycocyanin Suppress the HIV-1 Protease Activity

HIV-1 protease is an important enzyme that plays an imperative role in the maturation of the virus and consequently in the infection of new cells. It is the key enzyme of the HIV-1 life cycle acting at the post-entry level. As indicated in Table 3, the molecular interactions were examined by measuring residue contacts between HIV-1PR and C-PC residues, examining hydrogen bond and Van der Waals interactions, and examining binding energies. Briefly, a hydrogen bond was described as having a distance of <3.5 Å between the donor and acceptor with a donor-hydrogen acceptor angle >120°.

**Figure 5:**
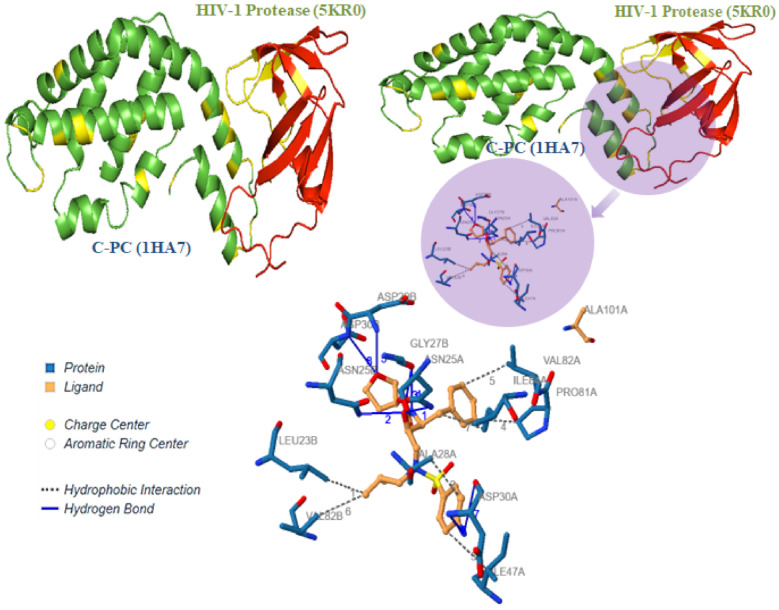
Molecular interactions between C-PC (1HA7) with HIV-1 Protease (5KR0). *In silico* molecular interactions between C-PC (green) and HIV-1-PR (red) are identified using protein–protein docking and simulations. Notably, active site and sub-site residues responsible for the proteolytic mechanism of HIV-1 PR are occupied by C-PC and hence, reduced HIV-1PR activity.

The in silico analysis of C-Phycocyanin and HIV-1 PR association predicted ample evidence of inhibitory interactions (Figure 5, Table 4). The proteolytic active site formed by amino acids Asp25, Asp29 and Asp30 in HIV-1PR is completely occupied by C-PC through hydrogen bonds, therefore inhibiting proteolysis. Additionally, C-PC also renders favorable conformational changes in the flexible glycine-rich ‘flap region (residues 44–57)’ consisting of two β-hairpins that covers the active cleft in HIV-1PR.

C-PC has made a noteworthy molecular interaction with HIV-1PR by forming total 08 hydrogen bonds with residues Asn25A, Asn25B, Gly27B, Asp29B, Asp30A, Asp30B and other Van der Waals interactions present in the active binding site of chain A and chain B (Table 4). Furthermore, the active binding site of HIV-1PR was lined up with the following active residues from chains A and B: Leu23, Thr26, Gly27, Ala28, Thr31, Val32, Lys45, Met46, Ile47, Gly48, Gly49, Ile50, Gly51, Phe53, Leu76, Thr80, Pro81, Val82, Asn83, and Ile84, where C-PC considerably made contacts (Figure 5 and Table 4). Similar types of interactions in active binding sites were also predicted using MOE tools (Table S1–S3 and Figure S2).

**Figure 6:**
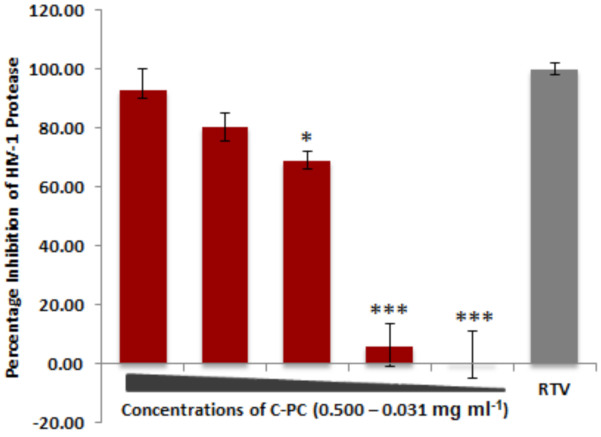
C-PC inhibits HIV-1 protease enzyme. The percentage inhibition of HIV-1 protease enzyme activity in the presence of C-PC in a dose-dependent (0.50–0.31 mg/mL) analysis compared to the known HIV-1 PR inhibitor RTV (10 μM). * *p* < 0.05 and *** *p* < 0.001.

The structural conformational changes rendered by C-PC in HIV-1PR structure through hydrogen bonds and Van der Waals interactions, which therefore led to inhibition of HIV-1PR activity. The potency of C-PC was examined against HIV-1 PR, in a dose-dependent manner, through in vitro assay (Figure 6). A maximum of 92.88% inhibition was observed at the highest C-PC concentration of 0.500 mg/mL (Figure 6). Furthermore, the C-PC showed the possession of a moderate *in vitro* inhibitory potency against HIV-1 PR with an IC_50_ value of 0.090 mg/mL. The results were validated with a known HIV-1 protease inhibitor Ritonavir (RTV) as a positive control with the IC_50_ value of 0.000005 mg/mL (data not shown).

### 3.6. In Silico Interactions of C-PC with Other Crucial HIV-1 Proteins and Viral Co-Receptor

As described in the methodology section, the interactions between C-PC (1HA7) and other HIV-1 proteins along with their receptors CXCR4 and CCR5 were also assessed using MOE software tools. A similar pattern of interaction and protocol of study was followed for these complexes vizHA7-1QS4 (integrase), 1HA7-37J0 (GP120), 1H7-3ODU (CxCR4) and 1HA7-4MBS (CCR5). The S-value −69.0002 and −79.1704 for HIV-1-INT and GP120, respectively, were found to be in noble interaction conformation with C-PC (Table S1). Glu109A of C-PC interacts with Arg199 of HIV-1-INT (1QS4) via a hydrophobic interaction with the distance of 3.375 Å, while the Met1B interacts with Asp202 with an ionic interaction. Only two interactions and a resulting S-value of −69.0002 pointed towards the weak interaction between C-PC and HIV-1-INT proteins. On the other hand, six various types of interactions (polar, hydrophobic, ionic, etc.) make C-PC and glycoprotein GP120 of HIV-1 (3J70) complex conformationally and energetically stable and profound. Polar interaction between Ala113A and Gln203 makes this protein –protein complex potently stable with a good energetic S-value of −79.1704 (Tables S2 and S3, Figures S3 and S4).

In the case of C-PC and CxCR4 complex, Glu7A, Gly114A, Ser14A, Asp109B, Glu117B, and Leu120B of C-PC protein, respectively, interacts with Lys1085, Arg1119, Leu1079, Arg1096, His140, and again His140 of CxCR4 protein. The S-value of −78.2342 for this complex suggests strong interaction of both the partner proteins. In the case of CCR5, the complex 1HA7-4MBS has shown the best S-value of −81.7884 with 1.2111 RMSD value and multiple interacting amino acids with ionic, hydrophobic and Van der Waals type of interactions. CxCR4 and CCR5 being the host receptors for viral entry, these also hold a significance to further verify in experimental settings (Tables S1–S3, Figures S5 and S6).

Overall, the protein– protein interaction profile of C-PC with HIV-1-GP120, co-receptor CxCR4 and CCR5 shows stronger interactions in comparison C-PC and HIV-1-INT complex.

### 3.7. ROS Scavenging Activity of C-Phycocyanin in HIV-1 Infected Cells

**Figure 7:**
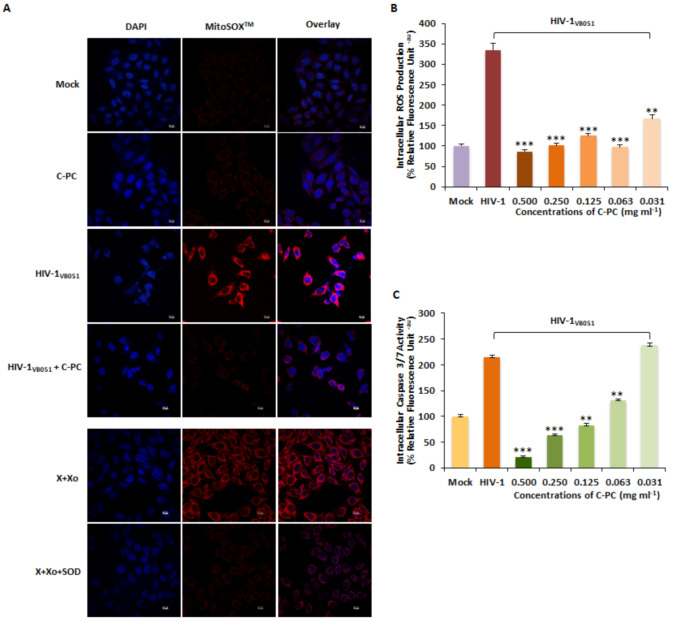
ROS Scavenging effects of C-PC in HIV-1-infected cells. (**A**) MitoSOX^TM^ Red Probe staining and ROS Scavenging effects of C-PC (0.1740 mg/mL–IC50 value) in HIV-1_VB051_-infected TZM-bl cells at 24 hpi. Mitochondria superoxide (red) and nuclear DNA (blue) showed under laser confocal microscope at standard magnification (63X 1.4 NA oil objective). The scale bar of the figure indicates a size of 50 µm. (**B**) Fluorescence measurement (arbitrary units, AU) was used to determine the ROS scavenging effects. Results are displayed as bar graphs that illustrate the proportion of fluorescence-positive cells labeled with MitoSOX^TM^ after 24 hpi. (**C**) Effect of C-PC on Caspases activation in HIV-1-infected TZM-bl cells. The figure represents the dose-dependent activity of caspase3/7 at 24 hpi. The data shown are the representative of at least three independent experiments. ** *p* < 0.01 and *** *p* < 0.001.

To determine the antioxidant activity of C-PC in HIV-1-infected TZM-bl cells, ROS level was measured using the MitoSOX fluorescent probe. Confocal microscopy results indicated higher levels of mitochondrial ROS content in the HIV-1-infected group compared to the mock-infected cells (Figure 7A). The C-PC treated HIV-1-infected cells showed lower fluorescence intensity (Figure 7A), which was further confirmed through the quantification analysis. The dose-dependent (0.500–0.031 mg/mL) treatment of C-PC significantly decreased the level of intracellular ROS up to 3.87-fold in HIV-1-infected cells suggesting that C-PC ameliorated the ROS production (Figure 7B). These results were further supported by the confocal microscopy using fluorescent-based molecular probe, 2,7 Dichlorodihydroflurescin diacetate (H2DCF-DA), where ROS elevation was detected in HIV-1-infected cells compared to mock infection, and C-PC treatment subsequently resulted in a reduction in intracellular ROS production (Figure S7).

We also examined the activation of caspase-3/7 to assess the effect of C-PC on the apoptotic cell death in HIV-1 infection. It was observed that there was a 2.15-fold increase in the activity of Caspase-3/7 in the HIV-1-infected cells compared with the mock infection. While the C-PC treatment (0.500–0.125 mg/mL) significantly inhibited the increased activation of caspase-3/7 in HIV-1-infected cells maximum by 10.29-fold up to 2.60-fold (Figure 7C).

**Figure 8:**
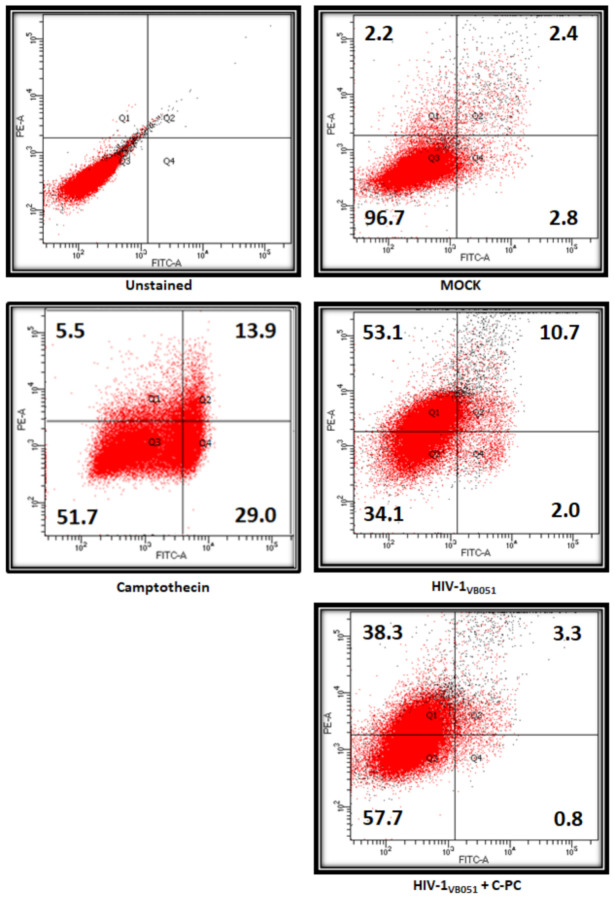
Annexin V-FITC Apoptosis staining. Representative flow cytometry dot plots illustrate annexin-FITC acquired on FL-1H (X-axis) versus PI staining acquired on FL-2H (Y-axis). HIV-1_VB051_-infected TZM-bl cells were treated with C-PC (0.1740 mg/mL) for 24 h and cell death was assessed using annexin V-fluorescein isothiocyanate (FITC)/propidium iodide (PI) staining represented as Q1: Necrotic cells, Q2: Late apoptotic cells, Q3: Live cells and Q4: Early apoptotic cells. TZM-bl cells treated for 6 h with Camptothecin (15 µm) was taken as a positive control in the experiment.

Furthermore, the effect of C-PC in HIV-1 induced cell death was investigated by annexin V/PI staining through FACS-based analysis. Results showed that cell death during HIV-1 infection was mainly due to necrosis (Q1) and apoptosis (Q2 and Q4). However, treatment of C-PC notably reduced both the necrosis and apoptotic population in HIV-1-infected cells (Figure 8). In HIV-1-infected population at 24 hpi, the cells were scattered in the different phases of cell death recorded as 2.0% of early apoptosis (Q4), 10.7% of late apoptosis (Q2), and 53.1% of necrosis (Q1); however, treatment of IC_50_ concentration (0.1740 mg/mL) of C-PC in HIV-1-infected TZM-bl cells considerably suppressed the distribution of dead cells by 60% in early apoptosis (0.8% in Q4), 69% in late apoptosis (3.3% in Q2), and 28% in necrosis (38.3% in Q1) phases (Figure 8). Camptothecin (15µm) was used as positive control of cell death due to apoptosis and necrosis.

## 4. Discussion

The limitations of antiretroviral medications in terms of resistance and toxicity becoming the greatest challenge for HIV-1 patients under ART; hence, the quest for new and powerful natural inhibitors is the only alternative solution against these major limitations. Therefore, in this present study, we examined the effect of such a natural inhibitor C-Phycocyanin or C-PC on HIV-1 extenuation. The results from our study could be briefed as C-PC significantly reduced viral infection and replication in wide clades of HIV-1 viruses (Figure 2). It also inhibited the important viral proteins, HIV-1 reverse transcriptase and protease, which is inferred from *in silico* molecular interactions (Figure 3 and Figure 5), as well as *in vitro* kit-based enzymatic validations (Figure 4 and Figure 6). Additionally, it has ROS scavenging activity indicating its potential application as an antioxidant agent (Figure 7), thus combined antioxidant effect and antiviral efficacy of C-PC may represent a promising therapeutic approach.

C-Phycocyanin, a phycobiliprotein that is present in some blue-green algae and marine cyanobacteria is known to exhibit a broad range of pharmacological actions [6,35,36]. Viral inhibitors produced from cyanobacteria and microalgae were reported to have antiviral potential throughout pathogenesis [37]. Through *in vitro* studies the anti-viral potential of Spirulina extract and its components have been mentioned previously, including the influenza virus, human immunodeficiency virus type 1 (HIV-1), herpes simplex virus type 1 (HSV-1), human cytomegalovirus, measles, and mumps virus infections [38,39,40,41,42]. It has also been reported that the extracts of Spirulina is completely safe and well tolerated through *in vitro* and *in vivo* studies [38]. Likewise, our findings support the previous studies by indicating that C-PC has anti-retroviral potential against various clades of HIV-1, and is safe up to 0.5 mg/mL to the cells, as confirmed by the cell viability assay.

Herein we focused on the inhibitory effects of C-PC protein against the HIV-1 reverse transcriptase and protease enzymes. The HIV-1-RT is a unique and essential enzyme that regulates the viral replication in the infected cells. Moreover, the HIV-1-RT performs a variety of tasks, including DNA polymerase and RNase H functions; while RNase H is responsible for the breaking down of viral RNA, DNA polymerase creates the cDNA from the viral RNA template, finally, contributing toward the HIV-1 replication cycle [43]. On the other hand, HIV-1-PR is another important enzyme that plays a crucial role in virus maturation process and consequently in the infection of new cells, by cleaving the polyproteins into functional viral proteins [44]. Hence, the HIV-1-PR is the key enzyme of the HIV-1 life cycle acting at the post-entry level. Due to their importance in viral life cycle, the HIV-1-RT and -PR are most often used for effective drug targets in HIV treatment [17]. The inhibitory effects of microalgae and cyanobacteria extracts on various other viral replications and their enzymatic activities have been elaborately discussed [45,46,47,48,49,50,51]. Our results also showed that C-PC served as potential HIV-1 inhibitors, both for HIV-1-RT as well as -PR. While the *in silico* analyses by computer-aid molecular docking predicted associations of C-PC with the HIV-1-RT and –PR, and potential interferences in their active sites to block the viral replication, the *in vitro* assays confirmed significant inhibition of both the HIV-1 enzyme activities in the presence of C-PC (0.05 mg/mL). The effect of C-PC on inhibition of HIV-1-RT and -PR was significant as compared to the effect of known chemical inhibitors NVP (RT) and RTV (PR), respectively. This study is the first to report the anti-HIV-1 activities of the phycobiliprotein C-PC against HIV-1-RT and -PR through *in silico* and *in vitro* approaches.

Several studies have revealed that the viral interactions with mitochondrial membranes and other components connected to mitochondria produce greater amounts of reactive oxygen species (ROS) [52]. These virus-induced mitochondrial ROS (mtROS) ultimately promotes viral replication through modulation of host pathways and covalent changes in the viral components, finally leads toward mtROS-induced apoptosis (intrinsic apoptosis), which is an important viral strategy for optimal intracellular viral replication and timely release of viral progeny [52]. The role of ROS in HIV pathogenesis was also speculated earlier [53,54]. Previous studies on phycobiliproteins have been reported for their antioxidant effects upon several pathogens and/or across the multiple cell types [55,56,57,58,59]. In our study, ROS generation was observed after 24 h of post-HIV-1 infection using molecular probe MitoSOX^TM^ and DCF-DA. HIV-1-infected cells showed increased fluorescence, while C-PC was found to reduce ROS generation in HIV-1-infected cells competently, ascertaining the role of C-PC protein as potent antioxidant. Hence, in agreement with the previous studies, C-PC also exhibited antioxidant effects, indicating its potential application as therapeutic agents against HIV-1 infection.

The loss of immune cells, notably CD4+ T-cells, is the defining feature of HIV-1 infection. Prior study showed that the HIV-1 envelope glycoproteins were responsible for the decrease of CD4+ T-cells, which resulted from oxidative stress accumulation and cell death [60]. According to these findings, managing the HIV-1 viral load and maintaining a healthy level of antioxidants are crucial components of HIV-1 patient care. Treatment with the antioxidant N-acetylcysteine in HIV-1-infected patients was able to increase glutathione levels and overall antioxidant status while lowering lipid peroxidation [61]. As ROS are short-lived and highly reactive molecules, high doses of ROS activate the mitochondria- and caspase-dependent apoptotic cell death signaling pathways. Caspase-3 acts as a point of convergence for both mitochondria-dependent and -independent processes in cells undergoing apoptotic cell death in response to oxidative stress [62,63]. Caspases, which are the members of the family of cysteine proteases, causes programmed cell death upon activation, and also found triggered by HIV-1 envelope proteins [64]. Our results revealed that HIV-1 virus-infected cells are responsible for the activation caspase-3/7, which is in line with the previous findings. However, C-PC supplementation in HIV-1-infected cells not only prevented the production of ROS but also decreased the activation of caspases-3/7 and the number of apoptosis cells, asserting its effect on cell death by reducing intracellular ROS.

Overall, this study identified the C-PC as a potential active substance that could obstruct HIV-1-RT and PR activities, therefore, offering an intriguing concept for developing natural anti-HIV drugs. However, it is plausible that additional anti-viral mechanisms could also be involved in C-PC mediated extenuation of HIV-1 replication, thus using the *in silico* analysis, ancillary molecular interactions between C-PC and other crucial viral proteins, including the glycoprotein GP-120, HIV-1 integrase and host targets like co-receptors CxCR4 and CCR5 was also investigated (Supplementary data). Although these interactions were not validated by the *in vitro* analysis, results from the *in silico* studies have indicated that C-PC also interacts with these viral proteins and co-receptors with appreciable binding interactions with significant bond energies between the amino acids of the associating proteins. Furthermore, undoubtedly the validation of anti-HIV-1 effects of the C-PC should be studied further. This will provide new insights into the possibility of using these proteins as adjunct therapy to restrict the spread of this deleterious virus.

## 5. Conclusions

In summary, we identified the negative regulatory effects of C-Phycocyanin on HIV-1 infection through *in silico* molecular associations with the viral proteins that has been further validated through *in vitro* mechanistic assays. The reverse transcriptase, which is the crucial enzyme for HIV-1 replication and the protease that plays the significant function in virus maturation and viral transmission, have been significantly suppressed the presence of C-PC. Moreover, the compound also exhibited ROS scavenging activity in HIV-1-infected cells, confirming its antioxidant potential and inhibitory effects on virus life cycle. Overall, our findings imply the potential antiretroviral property of C-Phycocyanin and might be an imperative asset in the field of anti-HIV medicine.

## Figures and Tables

**Table 1:** Interaction energy and Binding energies of HIV-1RT (1C0T) docked with C-PC (1HA7).

Parameters	HADDOCK Score	Cluster Size	RMSD Score	Van der Waals Energy	Electrostatic Energy	Desolvation Energy	Restraints Violation Energy	Buried Surface Area	Z-Score
Cluster 9	−58.3 ± 5.8	5	18.9 ± 0.0	−47.6 ± 0.6	−242.2± 16.1	13.8 ± 1.1	239.2 ± 57.1	1482.6 ± 25.7	−1.9
Cluster 1	−57.6 ± 2.7	45	20.7 ± 0.0	−50.2 ± 2.7	−131.8 ± 8.6	2.3 ± 0.4	166.3 ± 49.8	1637.4 ± 32.1	−1.8
Cluster 8	−48.8 ± 4.2	5	19.3 ± 0.2	−35.6 ± 3.6	−218.3 ± 13.0	6.7 ± 1.5	238.0 ± 43.1	1476.2 ± 25.2	−0.3
Cluster 6	−47.2 ± 4.7	6	17.7 ± 0.1	31.8 ± 1.8	−176.8 ± 13.1	−4.2 ± 3.3	241.6 ± 82.2	1363.2 ± 197.9	−0.0
Cluster 15	−45.2 ± 1.6	4	16.1 ± 0.1	−24.3 ± 1.1	−249.8 ± 5.3	−0.2 ± 1.7	293.7 ±16.3	1438.7 ± 29.9	0.3

**Table 2:** Hydrophobic and Hydrogen bond interaction details of HIV-1RT and C-PC.

**Hydrophobic Bond Interactions**									
**Index**		**Residue**		**AA**	**Distance**	**Ligand Atom**		**Protein Atom**	
1		95A		PRO	3.79	4907		585	
2		100A		LEU	3.79	4907		625	
3		100A		LEU	3.45	4909		624	
4		100A		LEU	3.81	4920		624	
5		106A		VAL	3.69	4917		693	
6		106A		VAL	3.34	4916		692	
7		181A		TYR	3.51	4910		1302	
8		188A		TYR	3.30	4912		1379	
9		318A		TYR	3.20	4918		2685	
**Hydrogen Bond Interactions**									
**Index**	**Residue**	**AA**	**Distance H-A**	**Distance D-A**	**Donor Angle**	**Protein Donor**	**Side Chain**	**Donor Atom**	**Acceptor Atom**
1	279A	LEU	3.03	3.66	125.22	✔	☓	2306 [Nam]	2318 [N3]
2	281A	LYS	3.42	4.01	118.54	✔	☓	2327 [N3]	2315 [O2]
3	281A	LYS	2.55	3.40	143.88	☓	☓	2318 [N3]	2327 [N3]

**Table 3:** Various interaction energy and Binding energies of HIV-1 PR (5KR0) docked with C-PC.

Parameter Cluster No.	HADDOCK Score	RMSD Score	Van der Waals Energy	Electrostatic Energy	Desolvation Energy	Restraints Violation Energy	Z-Score
Cluster 6	−83.1 ± 12.6	1.3 ± 0.9	−57.8 ± 4.8	−138.0 ± 16.7	−23.1 ± 6.5	254.1 ±44.92	−1.6
Cluster 5	−77.5 ± 9.9	15.0 ± 0.2	−30.8 ±3.6	−301.4 ± 65.8	−3.5 ± 2.4	170.9 ± 41.40	−1.1
Cluster 7	−74.7 ± 5.4	6.9 ± 0.7	−36.5 ± 5.7	−174.9 ±34.9	−13.9 ± 6.1	106.6 ± 23.59	−0.8
Cluster 2	−72.3 ± 4.2	10.4 ± 0.3	−48.7 ± 5.7	−92.3 ± 8.7	−18.9 ± 3.1	137.8 ± 24.64	−0.6
Cluster 1	−69.8 ± 8.5	11.0 ± 0.5	−50.2 ± 6.7	−104.7 ±16.7	−7.4 ± 1.7	88.1 ± 17.47	−0.4

**Table 4:** The protein–protein molecular interactions between HIV-1 PR and C-PC.

**Hydrophobic Bond Interactions**									
**Index**	**Residue**		**AA**	**Distance**		**Ligand Atom**		**Protein Atom**	
1	23B		LEU	3.97		1536		939	
2	28A		ALA	3.60		1531		215	
3	47A		ILE	3.95		1533		366	
4	81A		PRO	3.56		1524		616	
5	82A		VAL	3.71		1523		624	
6	82B		VAL	3.72		1536		1379	
7	84A		ILE	3.85		1519		640	
**Hydrogen Bond Interactions**									
**Index**	**Residue**	**AA**	**Distance H-A**	**Distance D-A**	**Donor Angle**	**Protein Donor**	**Side Chain**	**Donor Atom**	**Acceptor Atom**
1	25A	ASN	1.83	2.69	144.42	✔	☓	199 [Nam]	1543 [O3]
2	25B	ASN	2.50	3.27	134.51	☓	☓	955 [Nam]	1543 [O3]
3	27B	GLY	2.65	3.40	134.26	☓	☓	1543 [O3]	966 [O2]
4	27B	GLY	2.17	3.07	152.14	✔	☓	1538 [Nam]	966 [O2]
5	29B	ASP	3.27	3.72	109.53	✔	✔	972 [Nam]	1546 [O3]
6	30A	ASP	2.63	3.44	139.54	✔	☓	224 [Nam]	1540 [Npl]
7	30A	ASP	2.95	3.75	139.88	☓	☓	1540 [Npl]	227 [O2]
8	30B	ASP	2.49	3.41	156.75	✔	✔	980 [Nam]	1546 [O3]

## Data Availability

The data presented in this study are available on request from the corresponding author.

## Supplementary Materials

The following supporting information can be downloaded at: https://www.mdpi.com/article/10.3390/antiox11101942/s1. This section contains the supplemental text related to the detailed methods used involving the MOE software-based molecular docking and interaction studies. This section also contains all supplementary tables (Table S1–S3) and figures (Figure S1–S7), which are cited in the main text.
