# Supplementary material for: Elucidation of Antiviral and Antioxidant Potential of C-Phycocyanin against HIV-1 Infection through In Silico and In Vitro Approaches
Source: Antioxidants (Basel). 2022 Sep 28;11(10):1942. doi: 10.3390/antiox11101942 (PMC9598530; doi:10.3390/antiox11101942)
Supplement: Supplementary file 1 [file antioxidants-11-01942-s001.zip › Supplementary Data_Appendix A & B.pdf]

Supplementary Materials

Methods

Protein preparation and pocket finding of proteins of interest. The experimental structures of all proteins were obtained from protein data bank [1] and were prepared using Quickprep option of Molecular Operating Environment (MOE). This option cleans the protein, repairs any breaks or clashes and minimize the protein for further use. The coordinates for Light-Harvesting Phycobiliprotein, C-Phycocyanin (PDB ID: 1HA7) (the receptor) and other proteins to be docked with the receptor PDB available [PDB: 1C0T (HIV-1RT), 5KR0 (HIV-1PRO), 1QS4 (HIV-1INT), 3J70 (HIV-1GP120), 3ODU (CXCR4), and 4MBS (CCR5)] (ligand proteins) were processed in MOE software using the protein preparation function [2]. The programme added hydrogen atoms and partial charges. The recommended default settings for protonation were then used. The binding pocket of receptor protein (PDB ID: 1HA7) was found using pocket finder option of MOE.

Active site identification and grid generation. Ligand binding is a crucial step to work on for the treatment of various diseases. Non-specific ligand binding may show several artefacts in the body with higher toxic possibilities. The binding of ligand depend on several features such as H-bond donors and acceptors, hydro-phobic or -philic interaction, ionization, chelation of metal ions, etc. In our study, we used MOE pocket finder tool to find the binding site of our homology modelled protein. The best pocket found falls with-in the agreement of the active site of the protein based on available structural data. Receptor grid was generated after selection of the active site of protein.

Molecular docking of ligand proteins with receptor Phycocyanin. The protein–protein docking tool in MOE was used to create models of C-Phycocyanin containing the respective ligand protein complexes. The docking process began with a coarse-grained (CG) model, which reduced the computational search space, and exhaustive sampling was used to generate a set of initial poses. The Hopf fibration was used to generate a set of uniformly distributed rotations, and a Fast Fourier Transform (FFT) was used to sample all translations for a given rotation. This produced a set of initial docked poses for the models, which were then filtered based on restraints and fine-tuned. The refined model poses were then converted back to all atom models and refined further with side-chain packing and molecular mechanics minimization to produce a list of all atom protein–protein docked poses. Docked poses and rest figures were generated using YASARA view.

Two different computer-aid molecular docking systems have been used in this study; HADDOCK4.2 server and Molecular Operating Environment (MOE). Earlier, it has been reported that different tools may cause different predicted binding energies/sites for same compounds/ligands. Interestingly, the results of both molecular docking platforms were well comparable with a few additional interaction sites.

Bibliography

1. Berman, H.M.; Westbrook, J.; Feng, Z.; Gilliland, G.; Bhat, T.; Weissig, H.; Shindyalov, I.N.; Bourne, P.E. The Protein Data Bank. *Nucleic Acids Res.* **2000**, *28*, 235–242, doi:10.1093/nar/28.1.235.
2. Chemical Computing Group ULC Molecular Operating Environment (MOE) 2022. Available online: https://www.chemcomp.com/Products.htm (accessed on 7 June 2022).

Supplementary Tables

Table S1: Docking Energies of Protein-protein Interaction as Obtained from MOE

S.No.	Protein 1	Protein 2	S value (MOE docking score)	RMSD refine
1	**1HA7**	**1C0T (RTase) [1]**	**-80.38**	**1.105**
2	1HA7	1C0T	-79.69	0.8321
3	1HA7	1C0T	-74.37	1.585
1	**1HA7**	**5KR0 (Protease) [1]**	**-68.9273**	**1.1574**
2	1HA7	5KR0	-64.7816	0.7381
3	1HA7	5KR0	-64.4374	0.6055
1	**1HA7**	**1QS4 (Integrase) [1]**	**-69.0002**	**1.0446**
2	1HA7	1QS4	-66.1014	0.6809
3	1HA7	1QS4	-61.1322	0.9472
1	**1HA7**	**37J0 (GP120) [1]**	**-79.1704**	**0.3569**
2	1HA7	37J0	-77.2189	1.1570
3	1HA7	37J0	-75.9089	1.2039
1	**1HA7**	**3ODU (CxCR4) [1]**	**-78.2342**	**1.1768**
2	1HA7	3ODU	-78.0946	2.0183
3	1HA7	3ODU	-76.2096	0.8043
1	**1HA7**	**4MBS (CCR5) [1]**	**-81.7884**	**1.2111**
2	1HA7	4MBS	-76.3170	0.6275
3	1HA7	4MBS	-69.6705	0.4219

[1] **The best obtained values have been cited in the main text.**

Table S2: Protein-protein Docking Interaction between 1HA7 and HIV-1 Proteins or their Co-receptors

Interactions	Residue	Amino Acid	Distance	Interaction Type
**1HA7-1C0T** (C-PC vs Rtase)	Met69-A	Met230	3.591	Hydrophobic
	Gln70-A	Gly262	2.770	Polar
	Asp77-A	Lys259	4.456	Ionic
	Thr21-B	Arg448	4.841	Contact
**1HA7-5KR0** (C-PC vs Protease)	Asp108-A	Lys45	3.875	Ionic
	Asp116-A	Lys43	4.072	Van der Waals
	Thr118-B	Phe99	4.115	Van der Waals
	Ser14-A	Gly27	3.463	Hydrophobic
**1HA7-1QS4** (C-PC vs Integrase)	Glu109-A	Arg199	3.375	Hydrophobic
	Met1-B	Asp202	4.399	Ionic
**1HA7-3J70** (C-PC vs GP120)	Tyr110-A	Ala204	3.418	Hydrophobic
	Ala113-A	Gln203	2.601	Contact
	Tyr165-B	Lys322	3.575	Cation Π
	Gly114-B	Arg419	3.159	Van der Waals
	Glu117-B	Arg419	4.416	Ionic
	Arg168-B	Asn325	3.782	Hydrophobic
**1HA7-3ODU** (C-PC vs CxCR4)	Glu7-A	Lys1085	3.788	Ionic
	Gly114-A	Arg1119	3.296	Van der Waals
	Ser14-A	Leu1079	3.957	Hydrophobic
	Asp109-B	Arg1096	4.777	Ionic
	Glu117-B	His140	3.961	Hydrophobic
	Leu120-B	His140	3.324	Contact
**1HA7-4MBS** (C-PC vs CCR5)	Lys83-A	Asp1017	4.503	Ionic
	Arg79-A	Pro1020	3.631	Hydrophobic
	Gln78-A	Pro1026	3.562	Van der Waals
	Glu17-B	Arg230	4.663	Ionic
	Arg15-B	Lys229	3.739	Hydrophobic
	Ala22-B	Val142	2.651	Van der Waals

Table S3: Description of Interactions of Test Proteins with their Bound Ligand protein in the Experimentally solved PDB structures

Protein	Bound Ligand	Polar Interactions	Non-Polar Interactions
**1HA7** (C-Phycocyanin)	CYC [PHYCOCYANOBILIN]	Ala-75, Lys-83, Thr-66, Ser-78	Arg-79, Arg-57, Ile-118, Tyr-91, Leu-111, Tyr-76, Arg-86, Met-81, Tyr-90, Ile-88, Tyr-129, Cys-84, Leu-124, Trp-128, Val-59, Tyr-60
**1C0T** (Rtase)	BM1 [(R)-(+)9B-(3-METHYL)PHENYL-2,3-DIHYDROTHIAZOLO[2,3-A]ISOINDOL-5(9BH)-ONE]	Lys-103	Val-106, Tyr-188, Trp-229, Tyr-181, Pro-95, Leu-100
**5KR0** (Protease)	478 [{3-[(4-AMINO-BENZENESULFONYL)-ISOBUTYL-AMINO]-1-BENZYL-2-HYDROXY-PROPYL}-CARBAMIC ACID TETRAHYDRO-FURAN-3-YL ESTER)]	Asn-25, Asp-30, Gly-27, Gly-49	Val-82, Pro-81, Ala-28, Ile-47, Ile-50
**1QS4** (Integrase)	100 [1-(5-CHLOROINDOL-3-YL)-3-HYDROXY-3-(2H-TETRAZOL-5-YL)-PROPENONE]	Thr-66, Lys-156, Asp-64	NIL
**37J0** (GP120)	Not Reported [1]	-	-
**3ODU** (CxCR4)	ITD [(6,6-dimethyl-5,6-dihydroimidazo[2,1-b][1,3]thiazol-3-yl)methyl N,N'-dicyclohexylimidothiocarbamate]	NIL	Cys-186, Trp-94, His-113, Glu-288, Tyr-116
**4MBS** (CCR5)	MRV [4,4-difluoro-N-[(1S)-3-{(3-exo)-3-[3-methyl-5-(propan-2-yl)-4H-1,2,4-triazol-4-yl]-8-azabicyclo[3.2.1]oct-8-yl}-1-phenylpropyl]cyclohexanecarboxamide]	Thr-259, Thr-195, Tyr-251, Tyr-37	Leu-255, Phe-112, Ile-198, Phe-109, Tyr-108, Met-287, Trp-86, Tyr-89

[1] No ligand is found to be bound with GP120 protein

Supplementary Figures

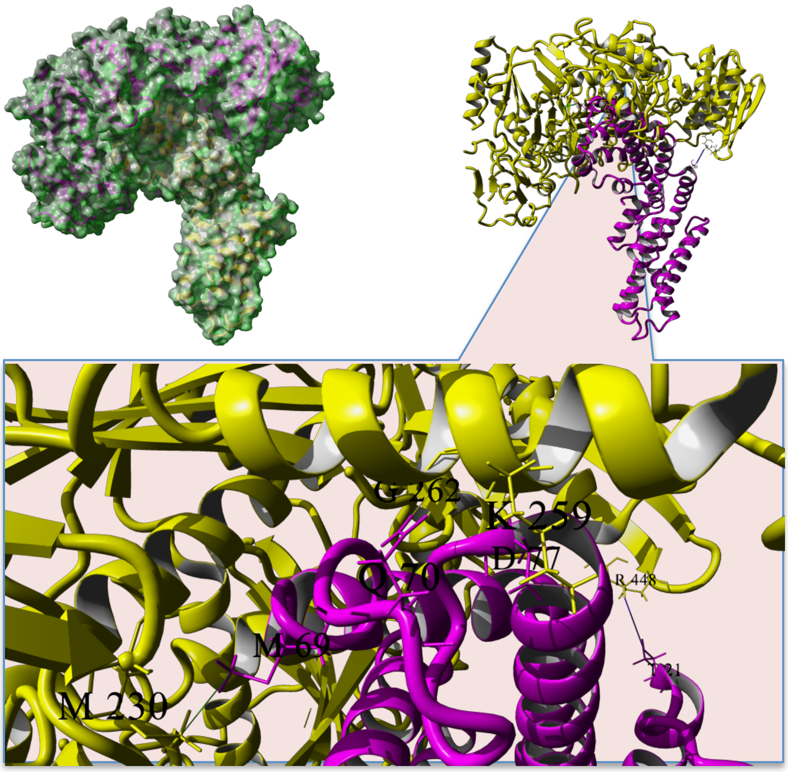

Supplementary Figure S1: ***Interaction between C-PC and HIV-1 Rtase.*** Conformational orientation of C-PC (1HA7 - magenta) with respect to the interacting HIV-1 RTase (1C0T - olive) shown in carton model (in left) and with surface representation (in right). Interactions between the two proteins are shown in the lower panel.

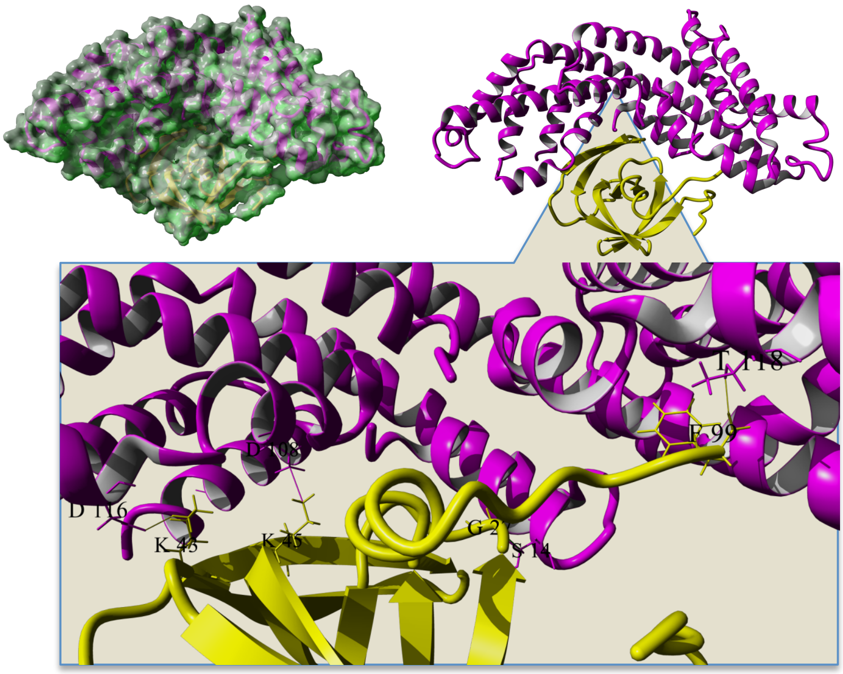

Supplementary Figure S2: ***Interaction between C-PC and HIV-1 Protease.*** Conformational orientation of C-PC (1HA7 - magenta) with respect to the interacting HIV-1 Protease (5KR0 - olive) shown in carton model (in left) and with surface representation (in right). Interactions between the two proteins are shown in the lower panel.

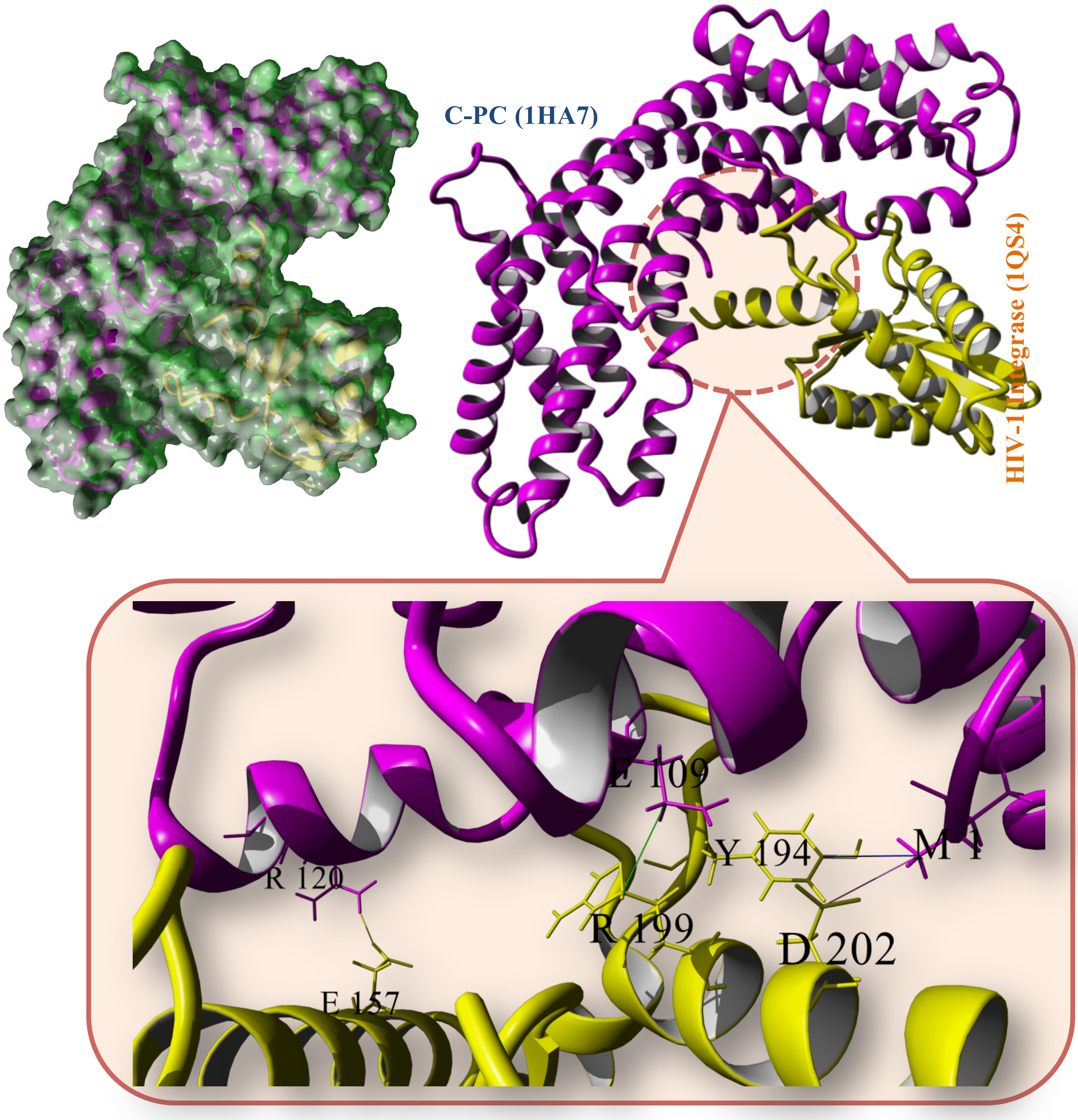

Supplementary Figure S3: ***Interaction between C-PC and HIV-1 Integrase.*** Conformational orientation of C-PC (1HA7 - magenta) with respect to the interacting HIV-1 integrase (1QS4 – olive green) shown with surface representation in carton model (top panel). Interactions between the two proteins are shown in the lower panel.

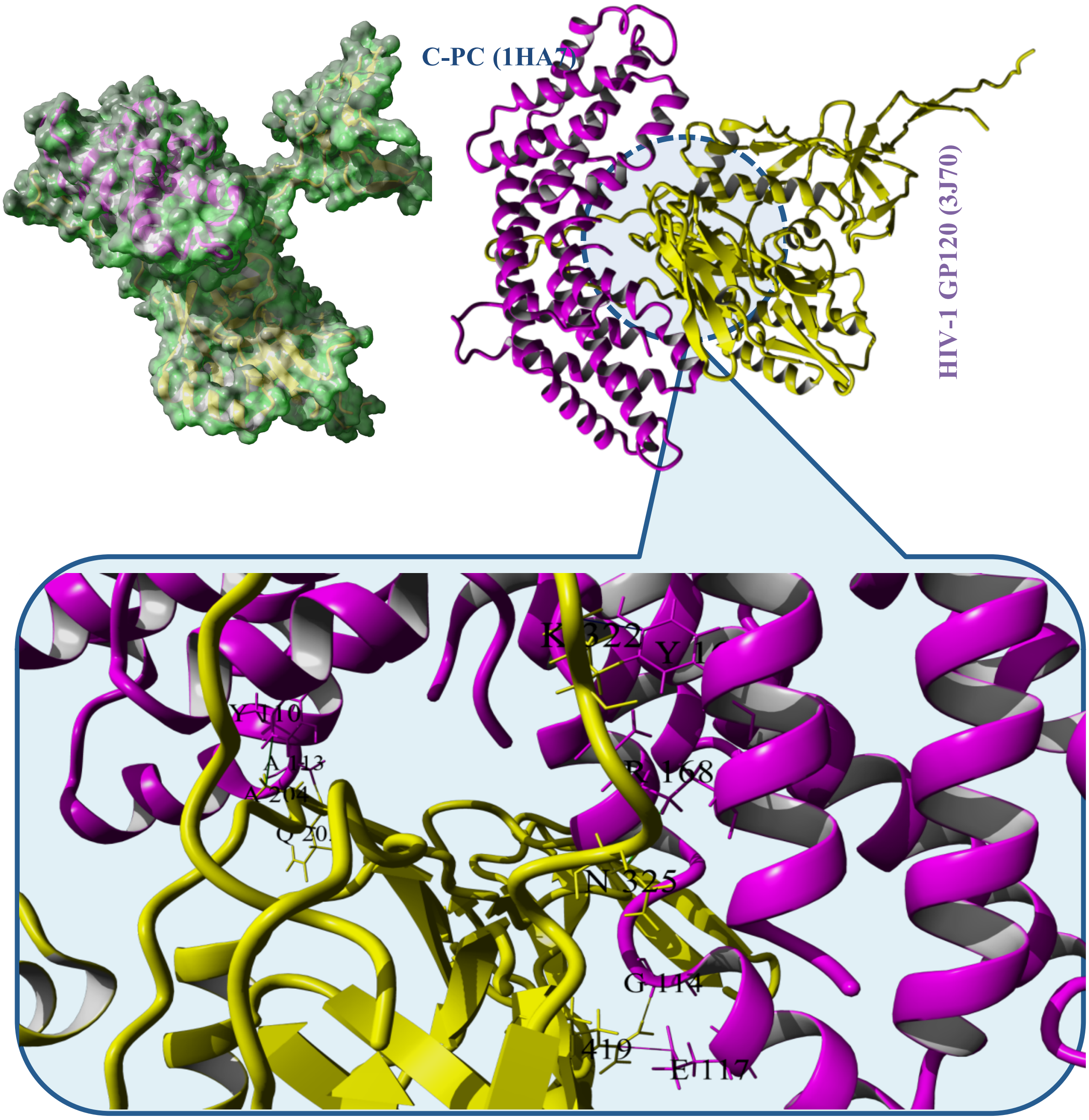

Supplementary Figure S4: ***Interaction between C-PC and HIV-1 Glycoprotein GP120.*** Conformational orientation of C-PC (1HA7 - magenta) with respect to the interacting HIV-1 GP120 (3J70 – olive green) shown with surface representation in carton model (top panel). Interactions between the two proteins are shown in the lower panel.

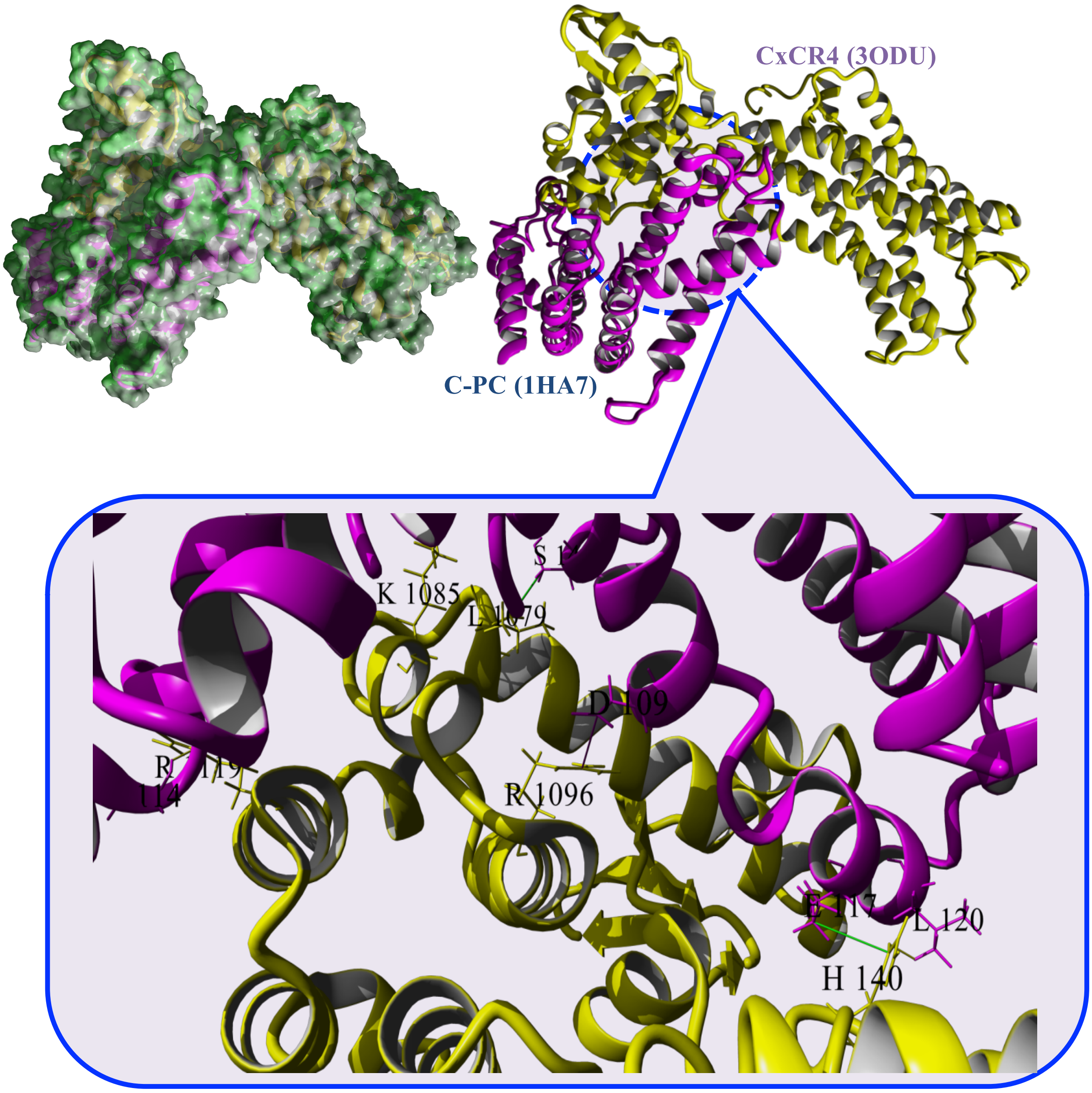

Supplementary Figure S5: ***Interaction between C-PC and HIV-1 Co-receptor CxCR4.*** Conformational orientation of C-PC (1HA7 - magenta) with respect to the interacting co-receptor CxCR4 (3ODU – olive green) shown with surface representation in carton model (top panel). Interactions between the two proteins are shown in the lower panel.

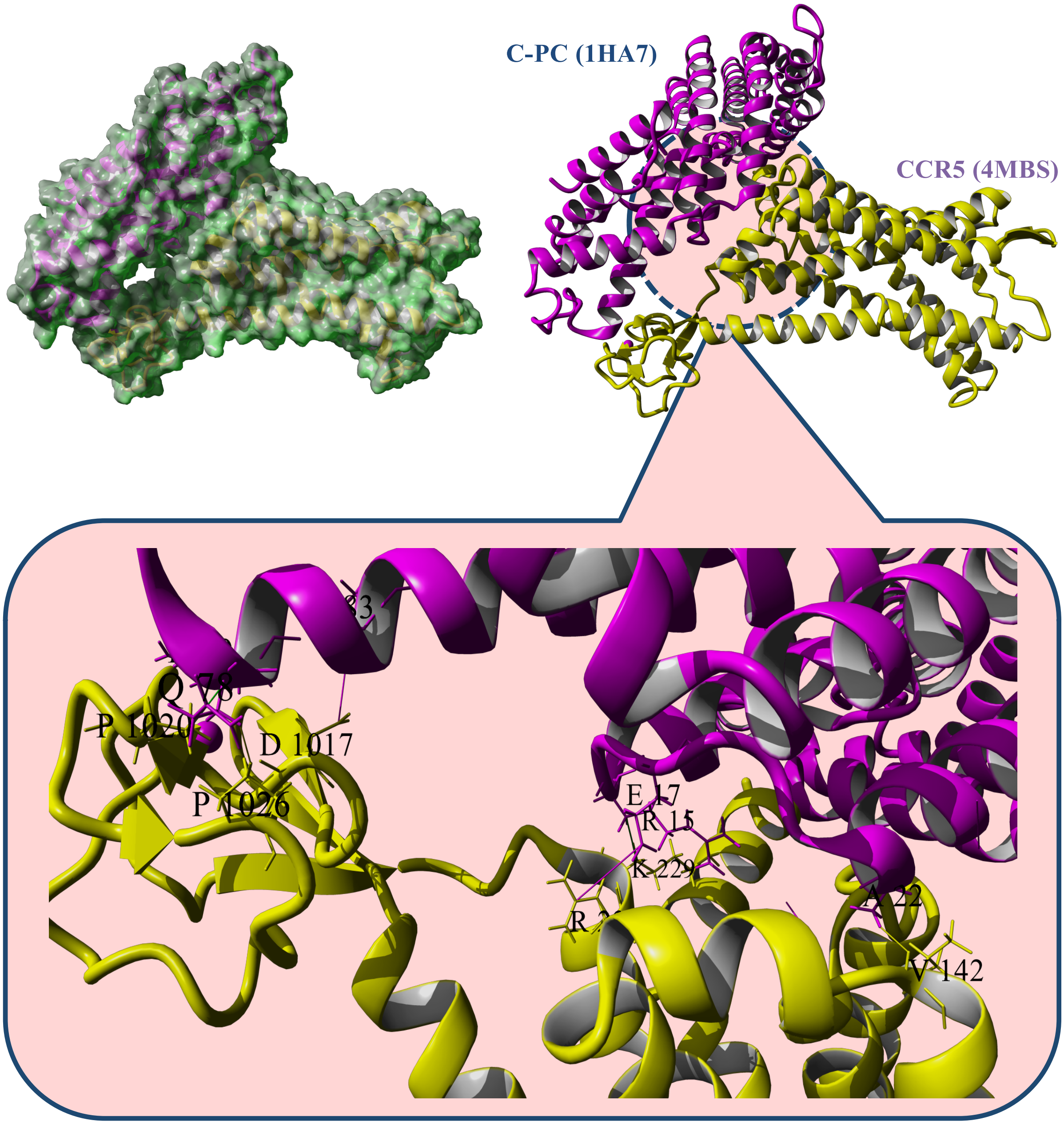

Supplementary Figure S6: ***Interaction between C-PC and HIV-1 Co-receptor CCR5.*** Conformational orientation of C-PC (1HA7 - magenta) with respect to the interacting co-receptor CCR5 (4MBS – olive green) shown with surface representation in carton model (top panel). Interactions between the two proteins are shown in the lower panel.

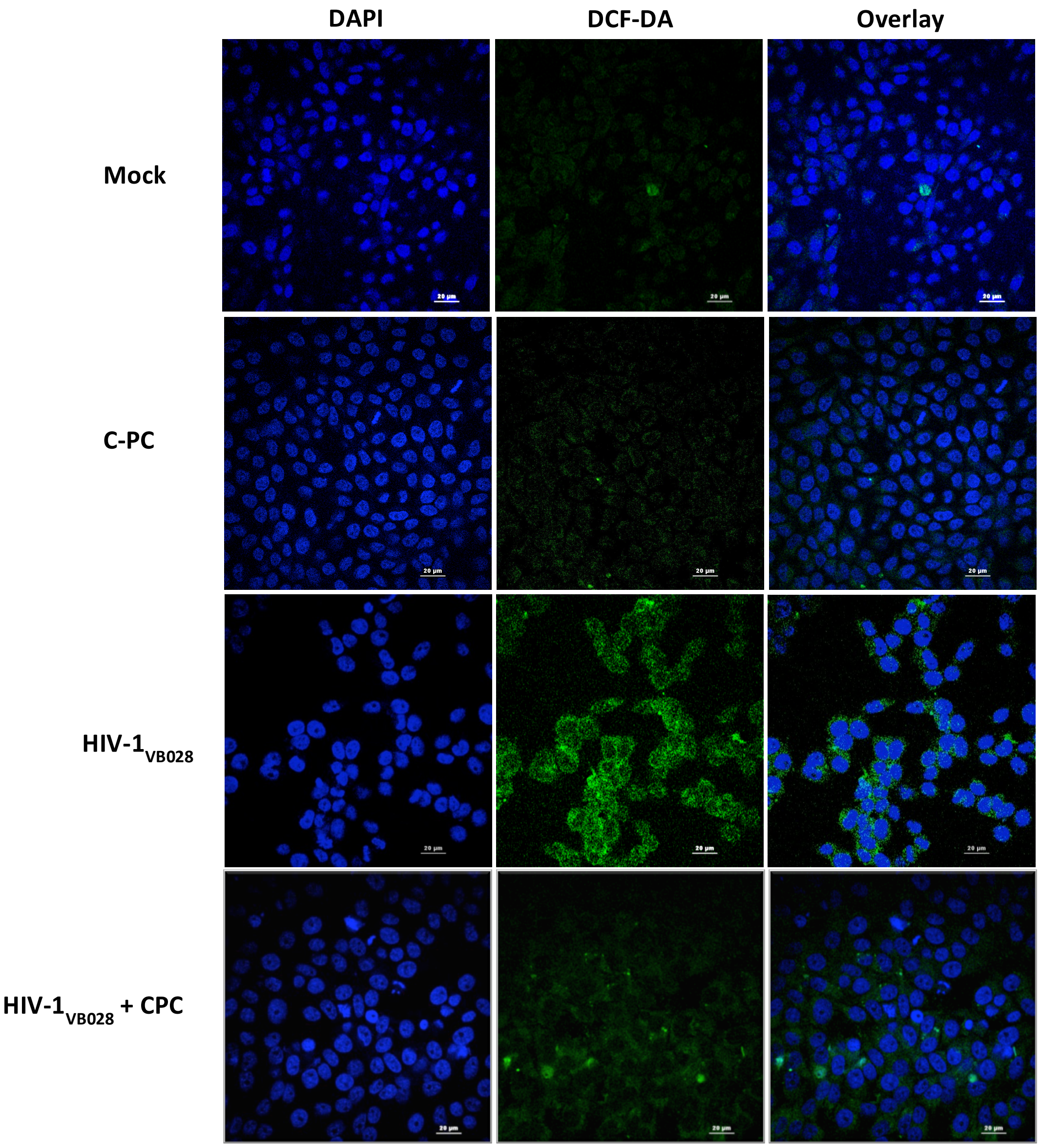

Supplementary Figure S7: *Confocal laser scanning microscope based ROS Scavenging effects of C-PC in DCF-DA-stained HIV-1 infected cells.*
